# Body mass index and participation in organized mammographic screening: a prospective cohort study

**DOI:** 10.1186/s12885-015-1296-8

**Published:** 2015-04-17

**Authors:** Sophie Sell Hellmann, Sisse Helle Njor, Elsebeth Lynge, My von Euler-Chelpin, Anja Olsen, Anne Tjønneland, Ilse Vejborg, Zorana Jovanovic Andersen

**Affiliations:** 1 Department of Public Health, University of Copenhagen, Øster Farimagsgade 5, 1014 Copenhagen, Denmark; 2 Danish Cancer Society, Institute of Cancer Epidemiology, Strandboulevarden 49, 2100 Copenhagen, Denmark; 3 Diagnostic Imaging Centre, Copenhagen University Hospital Rigshospitalet, Blegdamsvej 9, 2100 Copenhagen, Denmark

**Keywords:** Anthropometry, Body mass index, Body size, Obesity, Mammographic screening, Participation, Diabetes

## Abstract

**Background:**

Breast cancer is the leading cancer among women, and early diagnosis is essential for future prognosis. Evidence from mainly cross-sectional US studies with self-reported exposure and outcome found positive association of body mass index (BMI) with non-participation in mammographic screening, but hardly addressed the influence of potential effect-modifiers. We studied the association between objective measures of BMI and participation in mammographic screening in a Danish prospective cohort, and explored the influence of menopausal status, hormone therapy (HT), previous screening participation, and morbidities on this relationship.

**Methods:**

A total of 5,134 women from the Diet, Cancer, and Health cohort who were invited to population based mammographic screening in Copenhagen were included in analysis. Women were 50–64 years old at inclusion (1993–97) when their height and weight were measured and covariates collected via questionnaire. Odds ratios (OR) and 95% confidence intervals (CI) for the association between BMI and mammographic screening participation were estimated by logistic regression, adjusted for other breast cancer risk factors and morbidities. Effect modification was evaluated by an interaction term and tested by Wald test.

**Results:**

Underweight (BMI < 18.5 kg/m^2^, OR: 95% CI; 2.24: 1.27-3.96) and obese women of class II (BMI 35–40 kg/m^2^, 1.54: 0.99-2.39) and III (BMI ≥ 40 kg/m^2^, 1.81: 0.95-3.44) had significantly higher odds of non-participation than women with normal weight. This association was limited to postmenopausal women (Wald test p = 0.08), with enhanced non-participation in underweight (2.83: 1.52-5.27) and obese women of class II and III (1.84: 1.15-2.95; 2.47: 1.20-5.06) as compared to normal weight postmenopausal women. There was no effect modification by HT, previous screening participation, or morbidities, besides suggestive evidence of enhanced non-participation in diabetic overweight and obese women.

**Conclusions:**

Underweight and very obese postmenopausal women were significantly less likely to participate in mammographic screening than women with normal weight, while BMI was not related to screening in premenopausal women. Effect of BMI on mammographic screening participation was not significantly modified by HT, previous screening participation, or morbidities.

## Background

Breast cancer is the leading cancer type and cause of death from cancer among women in the Western world [1]. Diagnosis of breast cancer at an early stage is important for future prognosis [2]. Mammographic screening is an essential public health intervention in detecting early stage breast tumors, when treatment is more successful and survival more favorable [3]. High participation rate is paramount for the effectiveness of mammographic screening with participation rates above 70% being acceptable, and 75% desirable [3].

Obesity is positively associated with breast cancer risk in postmenopausal women [4], and possibly in premenopausal women when accounting for mammographic density [5]. Obesity is also related to poor breast cancer prognosis [6]. Recent reviews [7-9] of mainly cross-sectional studies suggested that obesity is associated with non-participation in mammographic screening, in particular among Caucasian women, but not among black American women. This implies that cultural differences in the perception of obesity seem to have an impact on their compliance with organized mammographic screening [7,8].

Current evidence on the relationship between body mass index (BMI) and participation in mammographic screening was mainly conducted in US populations, with high rates of opportunistic screening, and with profound socio-economic and health care access disparities that might confound the findings, since obesity is more prevalent among women with low socio-economic status [10]. Furthermore, existing studies mostly evaluated risk of non-participation based on self-reports of BMI and screening behavior, potentially masking effects due to recall and misclassification bias [11].

We studied an association between BMI and mammographic screening participation in a cohort of Danish women with objectively measured BMI and screening participation and with equal and free access to organized non-profit mammographic screening. We furthermore assessed whether menopausal status, previous mammographic screening participation, hormone replacement therapy (HT) use, or morbidities including stroke, myocardial infarction (MI), hypertension, hypercholesterolemia, or diabetes confounded or modified this association.

## Methods

### The Danish diet, cancer, and health cohort

The Danish Diet, Cancer, and Health cohort (DCH) is an associated cohort of the European Prospective Investigation into Cancer and Nutrition, described elsewhere [12]. Briefly, 79,729 women aged 50–64 years, born in Denmark, living in the large metropolitan areas of Copenhagen or Aarhus, and free of all cancer were invited, and 29,875 (37%) agreed to participate in the cohort [12]. Of total of 29,875 women in the DCH cohort, 21,154 lived in greater Copenhagen area, and less than a half of these lived in Copenhagen municipality (inner Copenhagen), where mammographic screening was in place since 1991 targeting women aged 50–69 years, and thus providing overlap with DCH cohort women, who were recruited between 1993 and 1999, when they were aged 50–65 years. Anthropometric measures were obtained by trained professionals at cohort baseline between 1993 and 1997, when also self-reported information on reproductive and life style exposures and morbidities were obtained via questionnaire. Measures of standing height and weight were recorded to the nearest 0.1 cm and 0.1 kg with participants wearing no shoes. BMI was calculated as weight divided by height in meters squared (kg/m^2^). BMI was defined according to the standard cutoff points by the World Health Organization (WHO) in categories of underweight: < 18.5, normal weight: 18.5-24.9, overweight: 25.0-29.9, obese class I: 30.0-34.9, obese class II: 35.0-39.9, and obese class III: > 40 kg/m^2^. Other covariates were self-reported and included menarche age, parity, age at first childbirth, breastfeeding, oral contraceptive use, HT use, menopausal status, menopausal age, education, smoking, alcohol use, and sports, described in Table 1. Self-reported morbidity with angina pectoris, diabetes, hypercholesterolemia, hypertension, MI, and stroke were defined as either having diagnoses or receiving medication for the specific disease. Premenopausal status was defined by no current hormone use and at least one menstrual bleeding within the last year. If information was not available on these variables, or if women had a hysterectomy with unknown age for menopause, then premenopausal status was defined by age ≤ 55 years at baseline. Postmenopausal status was defined by current HT use, bilateral oophorectomy, no menstrual bleeding within the last year and intact uterus, self-reported age of menopause, or age > 55 years, if information was not available on any of these variables.

**Table 1:** **Study population characteristics for 5,134 Danish women by BMI. Danish Diet, Cancer, and Health Cohort (1993–2008)**

		Body mass index^*^					
Characteristics	Total	Underweight	Normal	Overweight	Obese I	Obese II	Obese III
N women	5,134	74	2,381	1,772	657	182	68
N (%) non-participants in screening	557 (10.9)	17 (23.0)	285 (12.0)	152 (8.6)	62 (9.4)	28 (15.4)	13 (19.1)
N (%) Previously screened	3,914 (76.2)	59 (79.7)	1,750 (73.5)	1,387 (78.3)	517 (78.7)	149 (81.9)	52 (76.5)
Mean (SD) BMI, kg/m^2^	26.0 (4.7)	17.5 (0.9)	22.4 (1.6)	27.1 (1.4)	31.8 (1.3)	37.2 (1.4)	43.2 (3.6)
Mean (SD) birth cohort, year	1938 (4.5)	1936 (4.7)	1938 (4.5)	1937 (4.6)	1937 (4.5)	1937 (4.5)	1938 (4.6)
Mean (SD) age at screening, years	56.4 (4.5)	57.7 (4.6)	55.9 (4.4)	56.8 (4.5)	56.8 (4.5)	57.2 (4.4)	56.5 (4.5)
Mean (SD) age at menarche, years	13.6 (1.7)	14.1 (1.6)	13.8 (1.6)	13.5 (1.7)	13.3 (1.7)	13.4 (1.9)	13.1 (2.0)
Mean (SD) age at first birth, years	22.6 (4.2)	23.1 (4.0)	23.2 (4.1)	22.3 (4.2)	22.2 (4.4)	21.5 (3.7)	22.1 (3.7)
Mean (SD) age at menopause, years	48.4 (5.6)	45.9 (7.2)	48.5 (5.5)	48.4 (5.6)	48.6 (5.5)	49.0 (5.2)	47.4 (5.7)
N (%) basis school	1,194 (23.3)	14 (18.9)	460 (19.3)	438 (24.7)	206 (31.4)	57 (31.3)	19 (27.9)
N (%) higher education, 1–2 years	2,053 (40.0)	35 (47.3)	961 (40.4)	714 (40.3)	245 (37.3)	67 (36.8)	31 (45.6)
N (%) higher education, 3–4 years	1,228 (23.9)	15 (20.3)	586 (24.6)	424 (23.9)	144 (21.9)	46 (25.3)	13 (19.1)
N (%) higher education, ≥5 y.	659 (12.8)	10 (13.5)	374 (15.7)	196 (11.1)	62 (9.4)	12 (6.6)	5 (7.4)
N (%) postmenopausal	4,114 (80.1)	61 (82.4)	1,854 (77.9)	1,459 (82.3)	537 (81.7)	153 (84.1)	50 (73.5)
N (%) ever used HRT	2,097 (40.8)	32 (43.2)	976 (41.0)	775 (43.7)	245 (37.3)	54 (29.7)	15 (22.1)
N (%) nulliparous	549 (10.7)	15 (20.3)	269 (11.3)	183 (10.3)	59 (9.0)	15 (8.2)	8 (11.8)
N (%) 1–2 children	2,034 (39.6)	32 (43.2)	991 (41.6)	682 (38.5)	233 (35.5)	70 (38.5)	26 (38.2)
N (%) 3–4 children	1,925 (37.5)	19 (25.7)	882 (37.1)	683 (38.6)	248 (37.7)	72 (39.6)	21 (30.9)
N (%) ≥5 children	626 (12.2)	8 (10.8)	239 (10.0)	224 (12.6)	117 (17.8)	25 (13.7)	13 (19.1)
N (%) ever breastfed	4,228 (82.4)	53 (71.6)	1,945 (81.7)	1,494 (84.3)	538 (81.9)	150 (82.4)	48 (70.6)
N (%) ever used oral contraceptives	2,832 (55.2)	38 (51.4)	1,359 (57.1)	989 (55.8)	334 (50.8)	84 (46.2)	28 (41.2)
N (%) never smokers	1,913 (37.3)	16 (21.6)	796 (33.4)	688 (38.8)	297 (45.2)	86 (47.3)	30 (44.1)
N (%) current smokers	2,083 (40.5)	49 (66.2)	1,088 (45.7)	651 (36.7)	224 (34.1)	47 (25.8)	24 (35.3)
N (%) past smokers	1,138 (22.2)	9 (12.2)	497 (20.9)	433 (24.5)	136 (20.7)	49 (26.9)	14 (20.6)
N (%) alcohol abstainers	228 (4.4)	4 (5.4)	101 (4.2)	61 (3.4)	34 (5.2)	16 (8.8)	12 (17.6)
N (%) alcohol occasionally, monthly	1,607 (31.3)	23 (31.1)	645 (27.1)	559 (31.6)	261 (39.7)	89 (48.9)	30 (44.1)
N (%) alcohol ≤ 4 units/week	2,165 (42.2)	24 (32.4)	1,028 (43.2)	778 (43.9)	256 (39.0)	58 (31.9)	21 (30.9)
N (%) alcohol ≥ 5 units/week	1,134 (22.1)	23 (31.1)	607 (25.5)	374 (21.1)	106 (16.1)	19 (10.4)	5 (7.4)
N (%) participates in sport, weekly	2,543 (49.5)	29 (39.2)	1,251 (52.5)	892 (50.3)	276 (42.0)	69 (37.9)	26 (38.2)
N (%) angina pectoris	132 (2.6)	2 (2.7)	40 (1.7)	54 (3.0)	30 (4.6)	4 (2.2)	2 (2.9)
N (%) diabetes	92 (1.8)	0 (0.0)	25 (1.0)	25 (1.4)	28 (4.3)	8 (4.4)	6 (8.8)
N (%) hypercholesterolemia	307 (6.0)	4 (5.4)	113 (4.8)	116 (6.6)	53 (8.1)	16 (8.8)	5 (7.4)
N (%) hypertension	964 (18.8)	12 (16.2)	311 (13.1)	354 (20.0)	185 (28.2)	75 (41.2)	27 (39.7)
N (%) myocardial infarction	58 (1.1)	3 (4.0)	17 (0.7)	22 (1.2)	11 (1.7)	2 (1.1)	3 (4.4)
N (%) stroke	69 (1.3)	5 (6.8)	16 (0.7)	32 (1.8)	9 (1.4)	5 (2.7)	2 (2.9)

*Underweight (<18.5 kg/m^2^), normal weight (18.5-24.9 kg/m^2^), overweight (25.0-29.9 kg/m^2^), Obese class I (30.0-34.9 kg/m^2^), Obese class II (35.0-39.9 kg/m^2^), Obese class III (≥40.0 kg/m^2^). HRT – hormone replacement therapy; BMI – body mass index.

### Copenhagen mammographic screening program

Biennial mammographic screening was first introduced in Denmark in the municipality of Copenhagen in April 1991, free of charge to all women aged 50–69 years. Opportunistic mammographic screening is and was very limited in Denmark [13]. The central population register was used to define the target population for mammographic screening, contributing information on personal identification number (ID-number) issued to all residents of Denmark, migration, and vital status [14]. Targeted women were invited to screening if they: a) had not actively declined participation in previous screening rounds, b) did not have breast surgery within the past 18 months, c) were not bilateral mastectomized or had breast implants, where mammography was not technically possible [15]. A reminder was mailed to the women if they failed to respond to an invitation. The Copenhagen mammographic screening program performs well according to international prognostic indicators [16], with participation rate of 71% (1991–2001). We used data from the Copenhagen mammographic screening register from 1 April 1991 until 14 April 2008, containing ID-number for each women and bi-annual sequence of invitation dates, screening dates, history of reminders, and screening outcome. To facilitate prospective analyses of an effect of BMI on later participation in mammographic screening, we chose the first invitation date to screening after the DCH cohort baseline date (1993–1997). As women are invited to screening every two years, maximum time between cohort baseline and screening invitation was 2 years. The outcome was thus dichotomous indicator of non-participation (women who were invited but did not participate) and participation (women who were invited and attended mammographic screening). Furthermore, we defined previously screened (1) as women who participated in mammographic screening before cohort baseline (1993–97), and firstly screened (2) as women who did not participate in screening before cohort baseline, and have thus participated in mammographic screening for the first time after cohort baseline (1993–97).

### Statistical analysis

Logistic regression was performed to estimate the risk of non-participation with respect to BMI, with increasing level of adjustment for potential confounders: model 1: crude; model 2: adjusted for age and birth cohort (modeled as continuous covariates); model 3: further adjusted for menarche age, parity, age at first childbirth, breastfeeding, oral contraceptives, HT use, menopausal status, menopausal age, education, smoking, alcohol use, and sports (modeled as categorical covariates); model 4: further adjusted for angina pectoris, diabetes, hypercholesterolemia, hypertension, myocardial infraction, and stroke (modeled as categorical covariates). Effects are presented as odds ratios (OR) and 95% confidence intervals, with two-sided tests at the 5%-significance level. Chi-square trend test was further calculated to estimate potential dose–response effects across obese class I-III categories in a sensitivity analysis excluding underweight women. An effect modification of an association between BMI and screening participation with menopausal status, HT use, previous screening participation, and comorbidity was evaluated by introducing an interaction term and tested by Wald test. Data were analyzed using SAS version 9.2 (SAS Institute, Cary NC, USA). Informed consent was obtained from all study participants to use survey data and search information from medical registers^14^. The study was entirely a register based study approved by the Danish Data Inspection Agency by Danish law serving as ethical approval of register-based research.

## Results

Of 29,875 women in the DCH cohort, 21,154 lived in greater Copenhagen area, and of these, 7,507 who lived in Copenhagen municipality and fulfilled criteria, were invited to Copenhagen mammographic screening. Of these, 547 women were excluded as they were invited to screening only before DCH baseline (no invitations to screening after baseline) and 1,826 due to missing information on covariates, of these only 15 women were excluded due to missing data on BMI. Excluded 2,373 women did not differ from participating 5,134 women with respect to screening attendance, screening age, educational level, BMI, and smoking status, but were significantly more likely postmenopausal, parous, ever users of oral contraceptives and HT, and heavy drinkers (data not shown).

Of 5,134 women included in main analyses, 4,577 (89.1%) participated in screening (Table 1). Median BMI was 25.2 kg/m^2^, and mean time from measured BMI to mammographic screening invitation was 1.3 years (SD 1.5). Compared to normal weight women, obese women (class I-III) were less educated and more likely to be postmenopausal, never users of HT, parous, never smokers, alcohol abstainers, and physically inactive (Table 1).

The crude (Model 1) and fully adjusted OR (Model 4) for non-participation in mammographic screening for underweight as compared to women with normal BMI was 2.19 (1.26-3.82) and 2.24 (1.27-3.96), respectively (Table 2). Results were robust to adjustment for covariates, thus Model 4 is considered main model for which the results are presented in the paper. Overweight women were significantly more likely to participate in mammographic screening than women with normal weight with OR of 0.75 (0.61-0.93), as were women in obese category I, but not statistically significantly (0.85; 0.63-1.15). However, most obese women (class II and III) had a borderline significantly higher odds of non-participation of 1.54 (0.99-2.39) and 1.81 (0.95-3.44), respectively. There was a significant trend (p < 0.001) of increasing non-participation with increasing categories of obesity in sensitivity analysis excluding underweight women.

**Table 2:** **Association of BMI with mammographic screening non-participation in the Diet, Cancer, and Health Cohort**

			Model 1^1^	Model 2^2^	Model 3^3^	Model 4^4^
BMI	BMI value	N (%)	OR (95% CI)	OR (95% CI)	OR (95% CI)	OR (95% CI)
Underweight	<18.5	74 (1.4)	2.19 (1.26-3.82)	2.39 (1.37-4.18)	2.23 (1.27-3.95)	2.24 (1.27-3.96)
Normal range	18.5-24.9	2,381 (46.4)	1.00 (ref.)	1.00 (ref.)	1.00 (ref.)	1.00 (ref.)
Overweight	25.0-29.9	1,772 (34.5)	0.69 (0.56-0.85)	0.71 (0.58-0.87)	0.74 (0.60-0.92)	0.75 (0.61-0.93)
Obese class I	30.0-34.9	657 (12.8)	0.77 (0.57-1.02)	0.79 (0.59-1.06)	0.83 (0.62-1.12)	0.85 (0.63-1.15)
Obese class II	35.0-39.9	182 (3.6)	1.34 (0.88-2.04)	1.39 (0.91-2.12)	1.47 (0.95-2.27)	1.54 (0.99-2.39)
Obese class III	>40.0	68 (1.3)	1.74 (0.94-3.22)	1.80 (0.97-3.35)	1.76 (0.93-3.32)	1.81 (0.95-3.44)

^1^Model 1: Crude. ^2^Model 2: Adjusted for age and birth cohort. ^3^Model 3: Adjusted for age, birth cohort, education, menarche age, menopausal status, menopausal age, hormone replacement therapy use, oral contraceptives use, parity, age first childbirth, breastfeeding, smoking, alcohol, and sports.

^4^Model 4: Adjusted for model 3 + comorbidity.

Test for interaction between BMI and menopausal status was borderline significant (p = 0.08), indicating enhanced associations in post- and none in premenopausal women. Higher odds of non-participation in postmenopausal underweight (2.83; 1.52-5.27) and obese women of class II and III (1.84; 1.15-2.95 and 2.47; 1.20-5.06) were observed compared to postmenopausal women with normal weight (Table 3), with statistically significant trend (p = 0.0002) of increasing non-participation with increasing BMI. BMI was not associated with screening participation among premenopausal women (Table 3). There was no significant effect-modification by HT (p = 0.99), or by angina pectoris (p = 0.95), hypercholesterolemia (p = 0.86), hypertension (p = 0.48), myocardial infarction (p = 0.95), or stroke (p = 0.97). Interaction with previous screening participation was not statistically significant (p = 0.15), but indicated that non-participation in obese women was limited to those who previously participated in screening (overweight: 0.70; 0.54-0.91, obese class I: 1.01; 0.71-1.42, obese class II: 1.86; 1.14-3.04, and obese class III: 1.90; 0.91-3.96, all compared to normal weight), as compared to obese women participating in their first screening (overweight: 0.89; 0.60-1.30, obese class I: 0.47; 0.23-0.94, obese class II: 0.74; 0.24-2.34, and obese class III: 1.11; 0.29-4.35, all compared to normal weight). However, underweight women had high risk of non-participation in both, their first (2.09; 0.61-7.17) and subsequent screening (2.30; 1.19-4.45). Interaction with diabetes was not statistically significant (p = 0.34), due to small numbers, but diabetic overweight women had several fold higher odds of non-participation (overweight: 1.70; 0.12-23.94, obese class I: 4.38; 0.35-54.71, obese class II: 2.72; 0.10-74.66, and obese class III: 28.81; 1.37-604.02, all compared to normal weight) than overweight non-diabetic women (overweight: 0.74; 0.60-0.90, obese class I: 0.82; 0.61-1.11, obese class II: 1.58; 1.02-2.45, and obese class III: 1.58; 0.79-3.18, all compared to normal weight).

**Table 3:** **Association of BMI with mammographic screening non-participation by menopausal status in the Diet Cancer, and Health Cohort**

		Premenopausal women (n = 1,020)		Postmenopausal women (n = 4,114)	
BMI	BMI value	N (%)	OR (95% CI)^1^	N (%)	OR (95% CI)^1^
Underweight	<18.5	13 (1.3)	0.85 (0.18-4.15)	61 (1.5)	2.83 (1.52-5.27)
Normal range	18.5-24.9	527 (51.7)	1.00 (ref.)	1,854 (45.1)	1.00 (ref.)
Overweight	25.0-29.9	313 (30.7)	0.79 (0.51-1.23)	1,459 (35.5)	0.73 (0.57-0.93)
Obese class I	30.0-34.9	120 (11.8)	0.45 (0.20-0.99)	537 (13.0)	0.98 (0.70-1.37)
Obese class II	35.0-39.9	29 (2.8)	0.41 (0.09-1.92)	153 (3.7)	1.84 (1.15-2.95)
Obese class III	>40.0	18 (1.8)	0.63 (0.13-3.16)	50 (1.2)	2.47 (1.20-5.06)

^1^Adjusted for age, birth cohort, education, menarche age, hormone replacement therapy use, oral contraceptives use, parity, age at first childbirth, breastfeeding, smoking, alcohol, sports, and comorbidity.

## Discussion

We found lower mammographic screening participation among underweight and obese as compared to normal weight women with a dose–response relationship among obese class I-III. These associations were limited to postmenopausal women, and were robust to adjustment for other breast cancer risk factors and morbidities.

This is the first study on the association between BMI and mammographic screening participation in Denmark, where, as opposed to US, free, tax subsidized, and universal access to healthcare represents the core values in public health strategies, and where background rates of opportunistic mammographic screening are low. In Denmark, health care is free to all citizens, and mammographic screening is recommended by health authorities, and offered free of charge, with a personal invitation, to all women in age 50–69. In contrast, in US, health care is largely privatized and access and cost of mammographic screening differs by health insurance package a woman has, and if has health insurance. Thus, access to mammographic screening is different in Denmark and USA. Our study population consisted of a homogenous population of Caucasian women with equal and free access to health care services.

The results of our study add new insight to current evidence, which is mainly based on cross-sectional studies from US populations [17-30] with profound socio-economic and racial disparities in health care access, and high rates of mammographic opportunistic screening. Thus, results from US studies are vulnerable to selection bias and residual confounding from uncontrolled socio-economic barriers to screening. The only study conducted in a population with free and universal access to health care similar to the Danish health care model was a Canadian study by Mitchell et al. [26] who found no association between BMI and mammographic screening attendance. However, Mitchel et al. [26], as well as most other existing studies [17-25,27-30], relied on self-reports of BMI [17-25,27-30], which could attenuate associations, since obese women tend to underreport their actual weight [11,31,32].

The current study benefited from objectively measured BMI by health care professionals [12]. Only study by Chang et al. [18] in part used objectively measured BMI and chart extractions on mammographic screening participation in 2,832 women from the American Veterans Health Administration registry, and found no evidence of an association between BMI ≥ 30 and mammographic screening participation. Furthermore, majority of previous studies [17,19-24,26-30] were limited by self-reports on screening participation, where recall and misclassification bias cannot be ruled out [11].

We found a significant positive association between obesity and non-participation, in accordance with majority of existing studies, which were cross-sectional, assessing self-reported BMI typically to screening participation in last 2 years. We have furthermore, detected significant dose–response relationship between obesity and non-participation with a significant higher probability of non-participation with increasing obesity class, as reported earlier in some [19,22,24,25,27-30], but not all [17,18,20,23,26] studies. Cohen et al. [19] found a 30% significant increased risk of non-participation in mammographic screening among obese women of class III in 6,304 Caucasian women older than 42 years in the Southern Community Cohort Study. Ferrante et al. [22] found a 50% significantly increased risk of non-participation in mammographic screening in most obese of 7,544 women aged 40–74 years from the 2000 National Health Interview Survey. Wee et al. [29] found a 6-17% borderline significantly increased risk of non-participation in mammographic screening among obese women of class I-III in 5,277 Caucasian women aged 50–75 years from the 1998 National Health Interview Survey (NHIS). This finding was partly confirmed by Zhu et al. [30] in the 2000 NHIS, where a borderline significant 40% increased risk of non-participation in mammographic screening was found for the most obese (class III) of 7,692 Caucasian women aged 40–80 years, but failing to detect a dose–response relationship. Ostbye et al. [27] found a 27-40% significant increased risk of non-participation in mammographic screening among obese women (class I-III) in a cohort of 4,439 Caucasian women aged 50–64 years from the Health and Retirement Study.

The U-shaped association between BMI and mammographic screening participation, with high risk of non-participation in underweight and very obese women, was restricted to postmenopausal women only. BMI did not play a role in screening participation in premenopausal women. Cohen et al. [19] was the single study conducting analysis for effect-modification by menopausal status, and, in contrast to us, found no evidence of effect modification.

Mammographic screening attendance in underweight women has received less attention in literature than screening in overweight and obese women. Higher probability of morbidity in underweight compared to normal weight women might preclude underweight women from mammographic screening participation, which complicates unbiased estimation of their probability of nonparticipation in mammographic screening in relation to underweight. Few existing studies reported either no association with^27;29^ or increased non-participation in [17,20,24,30] mammographic screening among underweight women. Study by Chang et al. [18] found a significantly increased non-participation among underweight compared to normal weight in the Medicare population, whereas no association was found in the Veterans Health Administration population with objectively measured exposure and outcome information. In our study, we found a strong and statistically significant positive association between underweight and non-participation, even stronger than risk for non-participation related to obesity. The risk of nonparticipation in underweight women was not modified by previous screening participation. Furthermore, we found novel results that underweight was related to non-participation only in postmenopausal women, as no previous studies explored effect-modification by menopausal status in association between BMI and underweight.

The lower screening attendance among underweight and obese women could be a result of underlying illness and poorer general health than in women with normal weight, where absence from screening could be due to competing health burden and managing of existing disease. Morbidity with cardiovascular disease (angina pectoris, MI, hypertension, hypercholesterolemia) did not in our data explain absence from screening in underweight or obese women. There were no underweight women with diabetes in this study, but we have found two- to twenty- fold higher non-participation in mammographic screening among both overweight and obese diabetic, than in non-diabetic overweight and obese women. This finding indicates that diabetes may be an important barrier to screening, possibly stronger than obesity. Perhaps, diabetes may present competing health burden due to which diabetic women lack resources to go attend screening. These observations were based on limited number of cases, resulting in statistically insignificant p-value for interaction (0.34) and have never been reported before, and thus demand replication in other studies. However, in the face of obesity and diabetes epidemic worldwide, and being that obesity and diabetes both increase breast cancer risk and mortality [33,34], these findings indicate that targeted efforts in increased information about screening in diabetic women may have significant impact on their breast cancer prognosis.

Most of available evidence on association of mammographic screening participation with BMI is based on self-reported information on both exposure and outcome in cross-sectional design [34]. The study by Ostbye et al. [27] is only study with prospectively obtained self-reported BMI and screening information in-between two waves of cohort follow-up. Our study is thus to date the largest prospective cohort study presenting results on the association between objectively measured BMI exposure and subsequent mammographic screening participation.

Next to obesity, a complex mix of factors relating to health care systems, patients, and providers could determine if women abstain from mammographic screening [7,8]. A higher mammographic screening participation rate in rural compared to urban areas of Denmark and an age-adjusted U-shaped association between women’s educational level and socio-economic status and risk of non-participation was reported earlier [35-38]. Contrary to the US, Denmark has free access to health care services, including mammographic screening irrespective of socio-economic status, which is an established predictor for participation [8,39]. Cultural differences in the perception of obesity may also have an impact on women’s body-perception and compliance with organized mammographic screening [7,8]. Obesity rates are higher in USA than Denmark, and thus, perhaps general perception and acceptance of obesity, is higher in US, including health care system, which is likely more used to and prepared for dealing with obese persons. Still, obesity is observed to be related to non-participation in US and Denmark alike, possibly confirming earlier studies that the slim body ideal and negative perception of obesity in western cultures might predict non-participation in mammographic screening, in USA and Denmark alike [7,8]. A qualitative study by Friedman et al. [40] on weight barriers to mammographic screening proposed that negative attitudes and insensitive comments about weight from providers, medical equipment too small for obese, embarrassment of obesity during examination, fear of pain during examination, fear of cancer, competing demands on their time, and an impression of low breast cancer risk acted as possible barriers to screening in the studied population of obese women. We found that obese women, who previously participated in screening, seemed to have higher risk of non-participation in subsequent screening, indicating hypothetically that negative experience at a previous screening, such as pain or discomfort, may be a barrier for later participation. The same pattern was not observed for underweight women, who exhibited consistently high risk of non-participation in mammographic screening, unexplained by morbidities or previous participation in screening this study.

Since obesity is a predictor of breast cancer incidence, especially in postmenopausal women, breast cancer manifestation, and prognosis [6,41], it is important to identify barriers to mammographic screening [4]. Early detection by mammographic screening has been associated with improved prognostic tumor characteristics [16], and reduced morbidity and mortality from breast cancer [7,8,16,42]. Increased participation rates in mammographic screening among obese women, and possibly diabetic women, may therefore possibly be expected to result in reduced morbidity and mortality in this population.

Strength of our study is the prospective design with objectively measured BMI prior to register-based invitation to mammographic screening, in contrast to majority of existing studies of cross-sectional design with self-reports limiting causal inferences to determine causality. Objectively measured data on BMI and screening participation limit possibility of exposure misclassification, recall and information bias. As documented, DCH is not representative of the general population as participants had higher educational and socioeconomic status, and were likely more health awareness than non-participants [12]. Similarly, women in this study a higher participation of 89% as compared to an expected participation rate of 71% in Copenhagen in this period (1991–2001). Another major limitation was exclusion of 32% (2,373) of the eligible women (5,134) due to missing information on screening date or covariates. However, only 15 women were excluded for missing BMI measurements, and excluded 2,373 women did not differ from participating 5,134 women with respect to screening attendance, screening age, educational level, BMI, and smoking status, but were significantly more likely postmenopausal, parous, ever users of oral contraceptives and HT, and heavy drinkers (data not shown). As excluded women were not different from participating women with respect to BMI, our main exposure of interest, or screening participation, our outcome, missing data were not likely to pose major bias in our results on association between BMI and screening participation. Still, due to the fact that women in the study were more likely parous, heavy drinkers, and users of HT and OC than excluded women, the weakness is that our study sample may not be representative of the excluded women and general population. We used educational status as a proxy for socio-economic status, and educational status was an independent significant predictor for mammographic screening participation in our data. However, since ORs were robust to adjustment for all covariates in the multivariate models, further adjustment for socio-economic status would likely not impact the current results. Covariate information was based on self-reports, implying possible misclassification of covariate status. However, this misclassification would most likely be non-differential, since covariate data were obtained prospectively and independently of the information on the outcome of this study.

The higher probability of non-participation among underweight and obese women, and possibly in particular obese women with diabetes, could implicate later diagnosis, more advanced clinical stages of disease at diagnosis, and poorer morbidity and mortality [3]. Our findings could therefore have important public health implications, because obese post-menopausal women and diabetics have an established higher breast cancer risk [33,34], and since mammographic density, a strong predictor for breast cancer risk [43] inversely associated with BMI [4], tend to be higher among leaner women. Targeted information about screening in these groups of women may improve their breast cancer prognosis.

## Conclusions

Our study indicated a higher probability of non-participation among postmenopausal underweight and obese women as compared to women with normal weight, whereas no association was found for premenopausal women. Furthermore, we found a suggestive evidence of relevance of diabetes as a barrier to screening in overweight and obese women, although based on small numbers should be replicated in larger sized future studies.

## Abbreviations

BMI: Body mass index
DCH: Danish Diet, Cancer and Health cohort
WHO: The World Health Organization
HRT: Hormone replacement therapy
OR: Odds ratio
95% CI: 95% confidence interval
